# Microglial Activation, Tau Pathology, and Neurodegeneration Biomarkers Predict Longitudinal Cognitive Decline in Alzheimer’s Disease Continuum

**DOI:** 10.3389/fnagi.2022.848180

**Published:** 2022-06-30

**Authors:** Yi-He Chen, Rong-Rong Lin, Hui-Feng Huang, Yan-Yan Xue, Qing-Qing Tao

**Affiliations:** ^1^Department of Neurology and Research Center of Neurology in Second Affiliated Hospital, and Key Laboratory of Medical Neurobiology of Zhejiang Province, Zhejiang University School of Medicine, Hangzhou, China; ^2^Department of Neurology, Lishui Hospital, Zhejiang University School of Medicine, Lishui, China

**Keywords:** β-amyloid, brain atrophy, p-tau181, cognition change, sTREM2

## Abstract

**Purpose:**

Biomarkers used for predicting longitudinal cognitive change in Alzheimer’s disease (AD) continuum are still elusive. Tau pathology, neuroinflammation, and neurodegeneration are the leading candidate predictors. We aimed to determine these three aspects of biomarkers in cerebrospinal fluid (CSF) and plasma to predict longitudinal cognition status using Alzheimer’s Disease Neuroimaging Initiative (ADNI) cohort.

**Patients and Methods:**

A total of 430 subjects including, 96 cognitive normal (CN) with amyloid β (Aβ)-negative, 54 CN with Aβ-positive, 195 mild cognitive impairment (MCI) with Aβ-positive, and 85 AD with amyloid-positive (Aβ-positive are identified by CSF Aβ42/Aβ40 < 0.138). Aβ burden was evaluated by CSF and plasma Aβ42/Aβ40 ratio; tau pathology was evaluated by CSF and plasma phosphorylated-tau (p-tau181); microglial activation was measured by CSF soluble TREM2 (sTREM2) and progranulin (PGRN); neurodegeneration was measured by CSF and plasma t-tau and structural magnetic resonance imaging (MRI); cognition was examined annually over the subsequent 8 years using the Alzheimer’s Disease Assessment Scale Cognition 13-item scale (ADAS13) and Mini-Mental State Exam (MMSE). Linear mixed-effects models (LME) were applied to assess the correlation between biomarkers and longitudinal cognition decline, as well as their effect size on the prediction of longitudinal cognitive decline.

**Results:**

Baseline CSF Aβ42/Aβ40 ratio was decreased in MCI and AD compared to CN, while CSF p-tau181 and t-tau increased. Baseline CSF sTREM2 and PGRN did not show any differences in MCI and AD compared to CN. Baseline brain volumes (including the hippocampal, entorhinal, middle temporal lobe, and whole-brain) decreased in MCI and AD groups. For the longitudinal study, there were significant interaction effects of CSF p-tau181 × time, plasma p-tau181 × time, CSF sTREM2 × time, and brain volumes × time, indicating CSF, and plasma p-tau181, CSF sTREM2, and brain volumes could predict longitudinal cognition deterioration rate. CSF sTREM2, CSF, and plasma p-tau181 had similar medium prediction effects, while brain volumes showed stronger effects in predicting cognition decline.

**Conclusion:**

Our study reported that baseline CSF sTREM2, CSF, and plasma p-tau181, as well as structural MRI, could predict longitudinal cognitive decline in subjects with positive AD pathology. Plasma p-tau181 can be used as a relatively noninvasive reliable biomarker for AD longitudinal cognition decline prediction.

## Introduction

Alzheimer’s disease (AD) is the most common neurodegenerative disease characterized by progressive cognitive decline. Amyloid beta (Aβ) cascade and hyperphosphorylated tau (p-tau) hypothesis are considered as the major pathogenic mechanisms of AD (Long and Holtzman, 2019; Leng and Edison, 2021).

Except for Aβ and p-tau aggregation, neuroinflammation and microglial activation have been considered as the third pathological hallmark of AD (Kinney et al., 2018). Neuroinflammation and microglial activation even occurred at the early stage of AD (Femminella et al., 2019), and strongly correlated with AD pathology and disease progression (Fan et al., 2015; Jansen et al., 2019). The neuroinflammation can be quantified by specific cerebrospinal fluid (CSF) proteins or PET imaging with radioligands targeting translocator proteins (TSPO). For instance, ^11^C-PK11195-PET is extensively used for the neuroinflammation imaging study.

TREM2 is an AD susceptibility gene that is related to microglial activation. It is determined in genome-wide association studies analysis and confirmed by several subsequent studies (Jonsson et al., 2013; Jansen et al., 2019). The concentration of TREM2 is elevated in CSF of patients with AD and brain tissue of AD mouse models. Overexpression of TREM2 is related to the recruitment of activated microglia to amyloid plaques (Qin et al., 2021). Moreover, TREM2 is essential for microglial phagocytosis. TREM2 knockout leads to reduced microglial phagocytosis of β-amyloid, and an AD-related TREM2 variant (R47H) is associated with attenuated microgliosis (Ulrich et al., 2017). However, its role in microglial phagocytosis and Aβ clearance depends on the phase of AD (Jay et al., 2017). Soluble TREM2 (sTREM2) is a truncated TREM2 protein cleaved by ADAM (Schlepckow et al., 2017), making sTREM2 act as a biomarker of microglial activation and neuroinflammation. It can be detected both in CSF and plasma. Several studies have proposed the value of CSF sTREM2 in the diagnosis of AD (Suárez-Calvet et al., 2016; Rauchmann et al., 2019). CSF sTREM2 in AD seems to be dynamic depending on the disease progress (Liu et al., 2018; Ma et al., 2020). Progranulin (PGRN) is a secreted protein that is primarily expressed in neurons and microglia in the central nervous system. It demonstrates multifunctional biological functions, including vital roles in inflammation and neurodegeneration disease (Wang et al., 2021). PGRN deficiency promotes neuroinflammation, and it has been a valuable biomarker for frontotemporal lobar degeneration (FTLD).

However, biomarkers used for predicting longitudinal cognitive change in the AD continuum are still elusive. Among all the biomarkers, tau pathology, neuroinflammation (especially CSF sTREM2 and PGRN), and neurodegeneration are the leading candidate predictors of longitudinal cognitive impairment according to the previously reported studies but still need to be ascertained. Besides, their effect sizes on the prediction of longitudinal cognitive decline are unknown.

A better understanding of the biomarkers predicting a decline in AD will help to monitor disease progress and develop reliable prognostic and outcome measures in clinical practice. Here, we evaluated the role of microglial activation, tau pathology, and neurodegeneration in predicting the longitudinal cognitive deterioration in the AD continuum using the baseline assessments of CSF sTREM2, PGRN, p-tau181, t-tau, plasma p-tau, t-tau, and brain volumes.

## Materials and Methods

### Participants

All participants were enrolled in the Alzheimer’s Disease Neuroimaging Initiative (ADNI). In the ADNI database, the classifications of subjects [cognitive normal (CN), mild cognitive impairment (MCI), AD] were based on subjective memory complaints, Mini-Mental State Exam (MMSE) score, Clinical Dementia Rating score, and Wechsler Memory Scale–Revised score. In addition, subjects with MCI should be largely intact in general cognition and functional performance except for memory area and could not meet the diagnosis of dementia, while subjects with AD should meet the NINCDS-ADRDA diagnostic criteria for probable AD (Petersen et al., 2010). In ADNI GO and 2 cohorts, MCI population were divided into two subgroups, namely, early MCI (EMCI) and late MCI (LMCI). LMCI is defined as MCI in ADNI 1 cohort while EMCI show milder episodic memory impairment than classical LMCI subjects enrolled in ADNI 1 (Aisen et al., 2010). We then applied extra criteria to these subjects, including (1) subjects were from ADNI 1, GO and 2 cohorts; (2) subjects with baseline cognitive assessment and at least 1 year’s follow-up cognition scores; (3) subjects with baseline CSF biomarkers (including CSF sTREM2, PGRN, t-tau, p-tau181, and Aβ) and structural MRI data; and (4) subjects with positive Aβ pathology identified by CSF Aβ42/Aβ40 < 0.138 as described in previous studies (Guo et al., 2020; Korecka et al., 2020). CN subjects with negative Aβ pathology identified by CSF Aβ42/Aβ40 > 0.138. All of the subjects who met these criteria were included in the study.

Using these criteria, we finally selected 430 participants in this database, including 96 CN with Aβ-negative, 54 CN with Aβ-positive, 68 EMCI, and 127 LMCI with Aβ-positive, and 85 AD with Aβ-positive. These subjects were divided into four groups, namely, CN group with Aβ-negative (CN A-), CN group with Aβ-positive (CN A+), MCI group (EMCI+LMCI) with Aβ-positive (MCI A+), and AD group with Aβ-positive (AD A+).

### Cognitive Assessment

Cognition was examined annually in the baseline and the following 8 years using MMSE and Alzheimer’s disease Assessment Scale Cognition 13-item scale (ADAS13). As MMSE was a rudimentary assessment of cognition, we also used ADAS13 to evaluate the global cognition of each subject more precisely. Take note that not every subject had a complete record of an 8-year evaluation. The average number of visits and average follow-up years in each group are shown in Table 1. The number of cognitive measurements in every time points is shown in Supplementary Table 1. The data of every-year cognitive assessment are shown in Supplementary Table 2.

**TABLE 1:** Clinical features of CN group with amyloid-negative, CN, MCI, and AD groups with amyloid-positive.

	CN A^–^	CN A^+^	MCI A^+^	AD A^+^
Subjects	96	54	195	85
Age (mean ± sd)	73.9 ± 5.3	75.8 ± 6.0	72.7 ± 7.1	74.2 ± 8.4
**Gender**				
Male	58 (60.4%)	23 (42.6%)	114 (58.5%)	43 (50.6%)
Female	38 (39.6%)	31 (57.4%)	81 (41.5%)	42 (49.4%)
Years of education (mean ± sd)	16.4 ± 2.7	16.0 ± 2.0	15.9 ± 2.9	15.0 ± 2.6
*APOE* allele ε 4 carrier	10 (10.4%)	25 (46.3%)	131 (67.2%)	62 (72.9%)
Baseline ADAS13 score (mean ± sd)	9.23 ± 4.14	9.47 ± 3.84	18.12 ± 6.73	30.52 ± 7.53
Baseline MMSE score (mean ± sd)	29.1 ± 1.2	29.0 ± 1.1	27.4 ± 1.8	23.3 ± 1.8
Mean number of visits for ADAS13 (mean ± sd)	5.51 ± 1.92	5.57 ± 1.96	4.74 ± 1.94	2.64 ± 0.65
Mean follow-up years for ADAS13 (mean ± sd)	5.67 ± 2.32	5.48 ± 2.14	4.00 ± 2.21	1.67 ± 0.66
Mean number of visits for MMSE (mean ± sd)	5.52 ± 1.91	5.61 ± 1.96	4.80 ± 1.96	2.72 ± 0.68
Mean follow-up years for MMSE (mean ± sd)	5.65 ± 2.32	5.52 ± 2.15	4.06 ± 2.21	1.73 ± 0.68

### Magnetic Resonance Imaging Acquisition and Processing

T1-weighted MP RAGE was acquired for each subject at baseline view (1.5 T scanning for ADNI1 subjects and 3.0 T scanning for ADNI2/GO subjects). A 1.5 T data was run in FreeSurfer version 4.3, and 3.0 T data was run in FreeSurfer version 5.1. Each image was processed through several complicated steps for cortical reconstruction and volumetric segmentation (see “UCSF FreeSurfer Methods”). Then, hippocampal, entorhinal, and middle temporal lobe volume for each subject were calculated and adjusted for intracranial volume (ICV). These data are derived from UCSFFSX51_11_08_19.csv and UCSFFSX_11_02_15.csv.

### Cerebrospinal Fluid and Plasma Biomarkers Measurement

CSF sTREM2 and PGRN concentrations were measured by the ELISA protocol established by the Haass’ group in MSD platform (Capell et al., 2011; Suárez-Calvet et al., 2016). The data can be found in ADNI_HAASS_WASHU_LAB.csv file. CSF total tau and phosphorylated-tau181 were analyzed by electrochemiluminescence immunoassay following a Roche Studying Protocol in UPenn/ADNI biomarker Laboratory (Hansson et al., 2018), which were derived from UPENNBIOMK9_04_19_17.csv file. CSF Aβ42 and Aβ40 concentration were analyzed using 2D-UPLC tandem mass spectrometry, which was also widely used in measuring CSF biomarkers and its reproducibility and accuracy in measuring CSF Aβ42 and Aβ40 have also been confirmed (Korecka et al., 2014; Weber et al., 2019; Seino et al., 2021). Then, Aβ42/Aβ40 ratio was calculated for each subject and a published cut-off point of ratio (<0.138) was applied to identify positive amyloid deposition. CSF Aβ42 and Aβ40 concentration could be found in UPENNMSMSABETA.csv and UPENNMSMSABETA2.csv files. Plasma total Aβ42 and Aβ40 were analyzed by ABtest42 and ABtest40 briefly described as colorimetric tests based on ELISA (Perez-Grijalba et al., 2016). The results were derived from PLASMAABETA.csv file. Plasma p-tau181 were measured using single-molecule array (Karikari et al., 2020), where data could be found in UGOTPTAU181_06_18_20.csv file. Plasma tau was measured by the Single Molecule array (Simoa) technique and the Human total tau assay. And the data could be found in BLENNOWPLASMATAU.csv file.

### Statistical Analysis

CSF markers did not comply with normal distribution. Regional brain volume complying with normal distribution but did not show homogeneity of variance. As a result, Kruskal–Wallis test was used to evaluate differences in baseline biomarkers (CSF sTREM2, PGRN, Aβ42/Aβ40 ratio, p-tau181, and brain volumes) among CN, MCI, and AD groups. For pairwise comparison, we used the Wilcoxon rank-sum test with adjusted *p*-values using Holm adjustment. To test the correlation between baseline cognition score and baseline CSF biomarkers or brain volumes in cognitive impairments subjects, we conducted a multivariate linear regression model. For CSF sTREM2, PGRN, and brain volumes, diagnosis (EMCI, LMCI, or AD), age, gender, years of education, APOE genotype, CSF p-tau181, and CSF Aβ42/Aβ40 ratio were used as covariates. Moreover, for CSF p-tau181 (or Aβ42/Aβ40 ratio), only age, gender, years of education, APOE genotype, and CSF Aβ42/Aβ40 ratio (or CSF p-tau) were used as covariates. Then, we applied an LME regression model to identify whether baseline CSF sTREM2, PGRN, Aβ42/Aβ40 ratio, p-tau181, and brain volumes could predict cognitive decline. Diagnosis (CN, EMCI, LMCI, or AD), age, gender, education, APOE genotype, CSF p-tau181 × time, and Aβ42/Aβ40 × time were used as covariates with subject-specific intercepts and slopes (Ewers et al., 2019). The *p*-values of LME were adjusted using false discovery rate (FDR). All the statistical analyses were performed using R statistical software version 4.0.4. The *p*-values in LME models were acquired using the R package “lmerTes1”; the regression coefficients β in LME models were acquired using function “HLM_summary” in R package “bruceR”; the d scores in LME models were done using the R package “EMAtools.” A significance criterion was set to be *p* < 0.05.

## Results

### Clinical Features and Baseline Data

**FIGURE 1:**
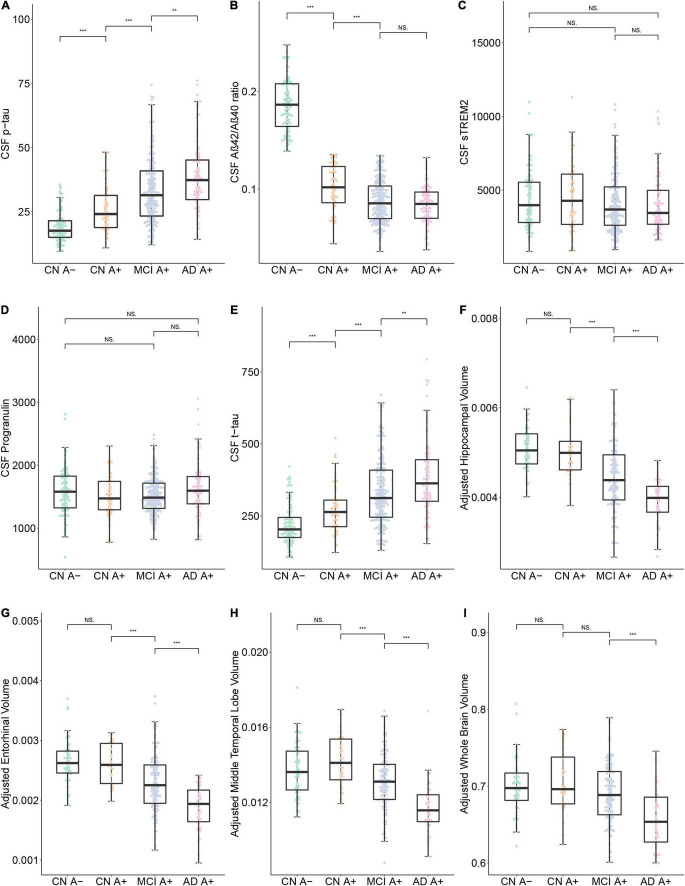
Baseline data comparison between CN A-, CN A+, MCI A+, and AD A+ groups. **(A–I)** Show the baseline Cerebrospinal Fluid (CSF) protein concentrations and specific brain region volumes in each group. Note that the brain volumes were computed using 3.0 T structural MRI. ***p < 0.001, **p < 0.01, *p < 0.05, ns, not significant.

The clinical features of each group are summarized in Table 1. There was a significant increase of CSF p-tau181 in the AD A+ and MCI A+ group compared to the CN A+ and CN A- groups (adjusted *p* < 0.001, respectively). We also observed an increased level of p-tau181 in the AD group compared to the MCI group (adjusted *p* = 0.003; Table 2 and Figure 1A). CSF Aβ42/Aβ40 ratio was decreased in MCI A+ and AD A+ groups compared to CN A+ and CN A- groups (adjusted *p* < 0.001, respectively). However, there was no difference in CSF Aβ42/Aβ40 ratio between MCI A+ and AD A+ groups (adjusted *p* = 0.23; Table 2 and Figure 1B). Baseline CSF sTREM2 and PRGN showed no significant difference among CN A–, CN A+, MCI A+, and AD A+ groups (*p* > 0.05; Table 2 and Figures 1C,D). AD A+ group had a higher baseline CSF t-tau than MCI A+, CN A+, and CN A- groups (adjusted *p* < 0.001, respectively; Table 2 and Figure 1E). Baseline-adjusted hippocampal volume was larger in CN A+ group than in MCI A+ and AD A+ group (adjusted *p* < 0.001, respectively), and CN A+ showed no difference with CN A-(Table 2, Figure 1F, and Supplementary Figure 1A). Meanwhile, entorhinal and middle temporal lobe volumes also showed similar tendencies among three groups (adjusted *p* < 0.001, respectively; Table 2, Figures 1G,H, and Supplementary Figures 1B,C), while whole-brain volume did not show any difference between CN A+ and MCI A+ groups (*p* = 0.19; Figure 1I and Supplementary Figure 1D).

**TABLE 2:** Baseline CSF sTREM2, progranulin, p-tau, t-tau concentration, Aβ42/Aβ40 ratio, and adjusted brain volume of CN group with amyloid-negative, CN, MCI, and AD groups with amyloid-positive.

	CN A^–^	CN A^+^	MCI A^+^	AD A^+^	*P*
sTREM2 (pg/mL)	3992.36 (2798.34)	4291.04 (3536.94)	3764.52 (2726.17)	3465.27 (2306.77)	0.42
progranulin (pg/mL)	1578.44 (525.12)	1471.59 (459.98)	1494.49 (422.83)	1594.63 (439.91)	0.26
t-tau (pg/mL)	203.7 (71.6)	264.0 (95.6)	314.2 (166.2)	363.4 (148.9)	<0.001
p-tau (pg/mL)	17.67 (6.73)	24.16 (13.07)	31.48 (17.83)	37.37 (16.05)	<0.001
Aβ42/Aβ40	0.187 (0.045)	0.102 (0.039)	0.085 (0.035)	0.085 (0.028)	<0.001
**HV (mm^3^)**					
1.5T	7224.69 ± 755.54	7319.12 ± 770.25	6247.05 ± 969.86	5800.33 ± 1108.60	
3.0T	7645.41 ± 862.99	7440.50 ± 767.24	6764.74 ± 1064.47	5898.88 ± 847.78	
**WBV (mm^3^)**					
1.5T	1012130.62 ± 82690.04	1004735.46 ± 91137.70	999603.62 ± 100589.75	962156.12 ± 128555.77	
3.0T	1055812.20 ± 96075.16	1045919.93 ± 104699.17	1055534.44 ± 105181.07	990730.29 ± 113365.74	
**EV (mm^3^)**					
1.5T	3919.88 ± 683.23	3720.85 ± 604.37	3244.77 ± 737.32	2755.47 ± 700.64	
3.0T	3981.96 ± 672.66	3853.18 ± 522.93	3510.52 ± 772.85	2827.69 ± 645.60	
**MTLV (mm^3^)**					
1.5T	20140.57 ± 2226.68	19510.58 ± 2707.68	18626.18 ± 2813.34	16847.00 ± 3598.37	
3.0T	20656.41 ± 2466.61	21021.75 ± 2546.80	19967.09 ± 2782.28	17602.71 ± 2869.59	
**Adjusted HV**					
1.5T	0.0047 ± 0.0005	0.0048 ± 0.0005	0.0040 ± 0.0006	0.0038 ± 0.0006	<0.001
3.0T	0.0051 ± 0.0005	0.0050 ± 0.0006	0.0044 ± 0.0007	0.0039 ± 0.0006	<0.001
**Adjusted WBV**					
1.5T	0.65 ± 0.04	0.66 ± 0.04	0.64 ± 0.04	0.63 ± 0.04	0.001
3.0T	0.70 ± 0.04	0.71 ± 0.04	0.69 ± 0.04	0.66 ± 0.04	<0.001
**Adjusted EV**					
1.5T	0.0025 ± 0.0005	0.0025 ± 0.0004	0.0021 ± 0.0005	0.0018 ± 0.0004	<0.001
3.0T	0.0026 ± 0.0004	0.0026 ± 0.0003	0.0023 ± 0.0005	0.0019 ± 0.0004	<0.001
**Adjusted MTLV**					
1.5T	0.0130 ± 0.0011	0.0129 ± 0.0013	0.0120 ± 0.0015	0.0109 ± 0.0015	<0.001
3.0T	0.0138 ± 0.0015	0.0142 ± 0.0014	0.0130 ± 0.0015	0.0117 ± 0.0014	<0.001

*Data complied to normal distribution were shown in mean ± SD, while data not complied to normal distribution were shown in median and interquartile. HV, hippocampal volume; EV, entorhinal volume; WBV, whole-brain volume; MTL, middle temporal lobe volume. Adjusted regional volume, regional volume/intracranial volume.*

### The Associations of Baseline Biomarkers and Cognition Status

**FIGURE 2:**
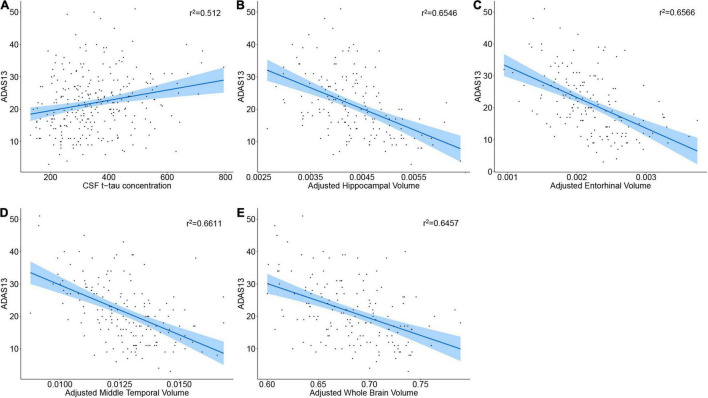
Correlation of baseline CSF proteins or brain volumes with cognition status. **(A–E)** The regression plot of CSF proteins or brain volumes. Note that the brain volumes were segmented from 3.0 T structural MRI.

To find whether higher baseline CSF p-tau, t-tau, lower CSF Aβ42/Aβ40 ratio, CSF sTREM2, PRGN, and lower brain volumes are associated with worse cognitive status, we computed a multivariate linear regression model in the cognitive impairment group (MCI A+ and AD A+). Subjects with higher CSF p-tau181 did not show higher ADAS13 score (*p* = 0.20, β = 0.04), and CSF Aβ42/Aβ40 ratio was not associated with ADAS13 score as well (*p* = 0.69, β = –8.09; Table 3). Interestingly, no relationship between baseline CSF sTREM2 or PGRN concentration and ADAS13 score was found (*p* = 0.70, β = –0.00085; *p* = 0.33, β = 0.00066, respectively; Table 3). Subjects with higher CSF t-tau level received a higher ADAS13 score (*p* = 0.00023, β = 0.06; Figure 2A and Table 3). Regional brain volumes played a positive role in baseline cognition where larger volume was associated with lower ADAS13 score (*p* < 0.05, respectively; Figures 2B–E, Supplementary Figures 2A–D, and Table 3).

**TABLE 3:** Correlations of baseline CSF p-tau, t-tau, Aβ42/Aβ40 ratio, sTREM2, PRGN, and brain volumes (segmented in 3.0 T MRI) with cognitive status (measured by ADAS13) in subjects with cognition impairment.

	*P*	Beta (SE)	*R* ^2^
CSF p-tau concentration	0.1998	4.188e-02 (3.259e-02)	0.49
CSF Aβ42/Aβ40 ratio	0.6906	–8.09229 (20.30536)	0.49
CSF sTREM2 concentration	0.6692	8.463e-05 (1.979e-04)	0.4903
CSF PRGN concentration	0.3292	6.638e-05 (6.792e-05)	0.4918
CSF t-tau concentration	0.00226	0.06351 (0.02415)	0.512
Adjusted HV	0.001413	–2.577e+03 (7.926e+02)	0.6546
Adjusted EV	0.000865	–3.0.115549e+03 (1.044e+03)	0.6566
Adjusted MTLV	0.000292	–1.200e+03 (3.238e+02)	0.6611
Adjusted WBV	0.012101	–34.04622 (13.40968)	0.6457

*The correlations CSF p-tau, t-tau, Aβ42/Aβ40 ratio, sTREM2, PRGN, and brain volume with cognitive status (measured by ADAS13) were analyzed using multivariate linear regression. Beta (SE) refers to standardized regression coefficient with standard error. R^2^ refers to coefficient of determination. HV, hippocampal volume; EV, entorhinal volume; WBV, whole-brain volume; MTL, middle temporal lobe volume.*

### The Role of Baseline Cerebrospinal Fluid P-Tau181 and Aβ42/Aβ40 in Predicting Longitudinal Cognitive Decline

**FIGURE 3:**
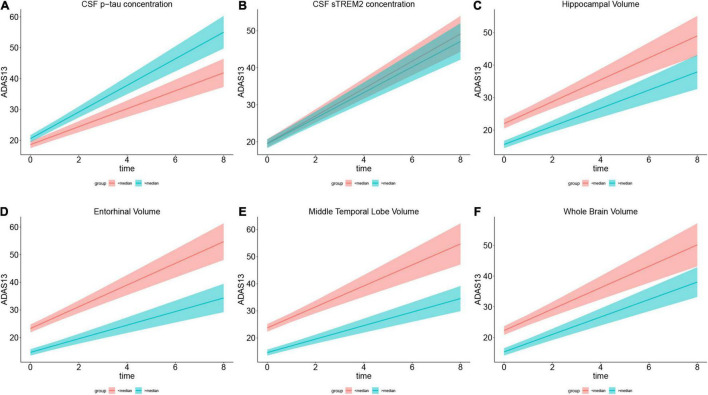
Role of each biomarker in cognitive change measured by ADAS13. **(A–F)** Show the annual ADAS13 change in cognitive impairment population with Aβ-positive. For directly illustration, each plot compares ADAS scores between the subgroup who scored below the median (in red) and the subgroup who scored above the median (in green) on the variable in the plot title. **(A)** Shows higher CSF p-tau concentration has faster cognitive decline in 8-year follow-up. **(B)** Illustrates higher CSF soluble TREM2 concentration is associated with slower cognitive change. **(C–F)** Show larger specific brain region volume (including hippocampal, entorhinal, and middle temporal and whole brain) is associated with slower cognitive decline. Brain volumes are segmented from 3.0 T structural MRI.

We used the LME model to analyze the correlation between baseline CSF p-tau181 and cognitive change in CN A-, CN A+, MCI A+, and AD A+ groups. The LME regression revealed that there was a significant interaction effect of CSF p-tau181 concentration × time in the MCI A+ combined AD A+ group (*p* = 0.001, β = 0.192) or in the MCI A+ group (*p* = 0.001, β = 0.22), but not in the AD A+ group (*p* = 0.77, β = 0.022; Table 4 and Figure 3A). In CN group (Aβ negative), the interaction effect of CSF p-tau181 × time showed no significant interaction effect (*p* = 0.63, β = 0.052; Table 4). Similar results were observed when using MMSE to evaluate cognition (*p* < 0.001, β = 0.248; Table 4 and Supplementary Figure 3A).

**TABLE 4:** Statistical analysis of linear mixed-effect regression for CSF Aβ42/Aβ40 and CSF p-tau concentration in amyloid positive (A+) groups alone or in combination.

CSF Aβ42/Aβ40	ADAS13	*P*	Adjusted *P*	Beta (SE)	*d*	MMSE	*P*	Adjusted *P*	Beta (SE)	*d*
CN A^–^		0.68	0.99	–0.030 (0.072)	NA		0.78	0.98	0.021 (0.076)	NA
CN A^+^		0.85	0.99	–0.023 (0.122)	NA		NA*	NA	NA	NA
MCI A^+^		0.99	0.99	0.001 (0.064)	NA		0.98	0.98	–0.002 (0.080)	NA
AD A^+^		0.50	0.99	0.053 (0.077)	NA		NA*	NA	NA	NA
MCI+AD A^+^		0.76	0.99	0.016 (0.054)	NA		0.28	0.84	–0.078 (0.072)	NA
**CSF p-tau**	**ADAS13**	***P***	**Adjusted *P***	**Beta (SE)**	***d***	**MMSE**	***P***	**Adjusted *P***	**Beta (SE)**	***d***
CN A^–^		0.50	0.63	0.052 (0.075)	NA		0.41	0.41	–0.066 (0.080)	NA
CN A^+^		0.01	0.02	0.319 (0.119)	0.84		NA*	NA	NA	NA
MCI A^+^		<0.001	0.001	0.220 (0.063)	0.56		<0.001	<0.001	–0.298 (0.079)	–0.63
AD A^+^		0.77	0.77	0.022 (0.073)	NA		NA*	NA	NA	NA
MCI+AD A^+^		<0.001	0.001	0.192 (0.052)	0.53		<0.001	<0.001	–0.248 (0.071)	–0.51

*p refers to the significance of interaction effect of CSF Aβ42/Aβ40 ratio (or CSF p-tau concentration) × time. The p-values are adjusted using false discovery rate (FDR). Beta (SE) refers to standardized regression coefficient with standard error. Cohen’s d refers to effect size of interaction effect of CSF Aβ42/Aβ40 ratio (or CSF p-tau concentration) × time. In ADAS13, negative effect size d means higher concentration (or ratio) is correlated with slower ADAS13 score increase, whereas positive effect size d means higher concentration (or ratio) is correlated with faster ADAS 13 score increase. In MMSE, negative effect size d means higher concentration (or ratio) is correlated with faster MMSE score decrease.*

*Means the model did not fit well.

Then, we applied the LME model to identify whether baseline CSF Aβ42/Aβ40 ratio could predict longitudinal cognitive deterioration. However, none of the group showed a significant CSF Aβ42/Aβ40 × time interaction effect (Table 4).

### The Role of Cerebrospinal Fluid sTREM2 and PRGN in Predicting Longitudinal Cognitive Decline

We applied the LME model to analyze the correlation of baseline CSF sTREM2 and cognitive change that was measured by ADAS13. The regression results reflected a significant interaction effect of baseline CSF sTREM2 and time (*p* < 0.001, β = –0.197) in the MCI+AD A+ population (Table 5 and Figure 3B). In CN group (either A- or A+), the correlation did not exist (*p* = 0.08, β = –0.158; *p* = 0.91, β = –0.053, respectively; Table 5). This significant interaction effect was likewise found in the MCI A+ group (*p* < 0.001, β = –0.22), whereas in the AD A+ group, we did not reach similar results (*p* = 0.97, β = –0.090; Table 5). We also applied the LME model to determine the correlation between baseline CSF sTREM2 and MMSE score, and the results were consistent with the above (Table 5 and Supplementary Figure 3B). However, baseline CSF PRGN was not associated with longitudinal cognitive change in cognitive impairment or CN subjects (Table 5).

**TABLE 5:** Statistical analysis of linear mixed-effect regression for CSF sTREM2 and CSF progranulin concentration in amyloid positive (A+) groups alone or in combination.

CSF sTREM2	ADAS13	*P*	Adjusted *P*	Beta (SE)	*d*	MMSE	*P*	Adjusted *P*	Beta (SE)	*d*
CN A-		0.05	0.08	–0.158 (0.080)	NA		0.53	0.53	0.056 (0.088)	NA
CN A+		0.73	0.91	–0.053 (0.150)	NA		NA*	NA	NA	NA
MCI A+		<0.001	<0.001	–0.220 (0.058)	–0.60		0.003	0.005	0.223 (0.074)	0.49
AD A+		0.97	0.97	–0.090 (0.076)	NA		NA*	NA	NA	NA
MCI+AD A+		<0.001	<0.001	–0.197 (0.049)	–0.58		0.001	0.003	0.225 (0.068)	0.49
**CSF PRGN**	**ADAS13**	***P***	**Adjusted *P***	**Beta (SE)**	***d***	**MMSE**	***P***	**Adjusted *P***	**Beta (SE)**	**Cohen’s d**
CN A-		0.46	0.58	–0.055 (0.075)	NA		0.27	0.70	–0.087 (0.078)	NA
CN A+		0.60	0.60	0.053 (0.100)	NA		NA*	NA	NA	NA
MCI A+		0.28	0.47	0.049 (0.045)	NA		0.70	0.70	–0.022 (0.056)	NA
AD A+		0.12	0.43	0.053 (0.085)	NA		NA*	NA	NA	NA
MCI+AD A+		0.17	0.43	0.056 (0.041)	NA		0.58	0.70	–0.031 (0.056)	NA

*p refers to the significance of interaction effect of CSF sTREM2 concentration (or CSF progranulin) × time. The p-values are adjusted using false discovery rate (FDR). Beta (SE) refers to standardized regression coefficient with standard error. Cohen’s d refers to effect size of interaction effect of CSF sTREM2 concentration (or CSF progranulin) × time. In ADAS13, negative effect size d means higher concentrations are correlated with slower ADAS13 score increase, whereas positive effect size d means higher baseline concentrations are correlated with faster ADAS13 score increase. In MMSE, positive effect size d means higher concentrations are correlated with slower MMSE score decrease.*

*Means the model did not fit well.

### The Role of Cerebrospinal Fluid T-Tau and Brain Atrophy in Predicting Longitudinal Cognitive Decline

We assessed the association between CSF t-tau and cognitive decline. By computing a LME model, CSF t-tau × time showed no significant interaction effect in cognitive impairment group and CN group (Table 6).

**TABLE 6:** Statistical analysis of linear mixed-effect regression for CSF t-tau concentration and specific brain region volumes (segmented in 3.0 T structural MRI) in amyloid positive (A^+^) groups alone or in combination.

CSF t-tau	ADAS13	*P*	Adjusted *P*	Beta (SE)	d	MMSE	*P*	Adjusted *P*	Beta (SE)	d
CN A^–^		0.63	0.84	0.199 (0.409)	NA		NA*	NA	NA	NA
CN A^+^		0.12	0.60	–0.816 (0.514)	NA		NA*	NA	NA	NA
MCI A^+^		0.71	0.84	–0.106 (0.288)	NA		0.84	0.84	–0.074 (0.356)	NA
AD A^+^		0.61	0.84	0.166 (0.325)	NA		NA*	NA	NA	NA
MCI+AD A^+^		0.84	0.84	0.046 (0.235)	NA		0.12	0.24	–0.491 (0.312)	NA
**Adjusted HV**	**ADAS13**	***P***	**Adjusted *P***	**Beta (SE)**	***d***	**MMSE**	***P***	**Adjusted *P***	**Beta (SE)**	***d***
CN A^–^		0.89	0.89	–0.012 (0.085)	NA		0.10	0.10	0.195 (0.116)	NA
CN A^+^		0.58	0.73	0.083 (0.148)	NA		NA*	NA	NA	NA
MCI A^+^		0.02	0.04	–0.160 (0.065)	–0.53		0.001	0.002	0.307 (0.091)	0.70
AD A^+^		0.17	0.28	0.148 (0.104)	NA		NA*	NA	NA	NA
MCI+AD A^+^		0.01	0.04	–0.145 (0.057)	–0.52		<0.001	<0.001	0.366 (0.086)	0.91
**Adjusted EV**	**ADAS13**	***P***	**Adjusted *P***	**Beta (SE)**	***d***	**MMSE**	***P***	**Adjusted *P***	**Beta (SE)**	***d***
CN A^–^		0.67	0.81	0.034 (0.080)	NA		0.04	0.04	0.232 (0.109)	NA
CN A^+^		0.81	0.81	–0.152 (0.148)	NA		NA*	NA	NA	NA
MCI A^+^		0.008	0.02	–0.199 (0.073)	–0.65		0.000231	0.000347	0.377 (0.097)	0.89
AD A^+^		0.51	0.81	0.069 (0.102)	NA		NA*	NA	NA	NA
MCI+AD A^+^		0.002	0.008	–0.206 (0.063)	–0.73		<0.001	<0.001	0.411 (0.092)	1.1
**Adjusted MTLV**	**ADAS13**	***P***	**Adjusted *P***	**Beta (SE)**	***d***	**MMSE**	***P***	**Adjusted *P***	**Beta (SE)**	**d**
CN A^–^		0.49	0.6125	–0.057 (0.082)	NA		0.54	0.54	0.072 (0.117)	NA
CN A^+^		0.93	0.93	–0.010 (0.121)	NA		NA*	NA	NA	NA
MCI A^+^		0.01	0.03	–0.158 (0.062)	–0.54		<0.001	<0.001	0.321 (0.086)	0.79
AD A^+^		0.10	0.17	–0.176 (0.102)	NA		NA*	NA	NA	NA
MCI+AD A^+^		<0.001	0.002	–0.205 (0.055)	–0.72		<0.001	<0.001	0.449 (0.081)	1.1
**Adjusted WBV**	**ADAS13**	***P***	**Adjusted *P***	**Beta (SE)**	***d***	**MMSE**	***P***	**Adjusted *P***	**Beta (SE)**	***d***
CN A^–^		0.77	0.87	0.026 (0.086)	NA		0.21	0.21	0.153 (0.120)	NA
CN A^+^		0.78	0.87	–0.041 (0.144)	NA		NA*	NA	NA	NA
MCI A^+^		0.008	0.02	–0.188 (0.069)	–0.62		<0.001	<0.001	0.417 (0.092)	1.0
AD A^+^		0.87	0.87	0.016 (0.099)	NA		NA*	NA	NA	NA
MCI+AD A^+^		<0.001	0.004	–0.207 (0.059)	–0.72		0.005	<0.001	0.499 (0.084)	1.3

*p refers to the significance of interaction effect of CSF t-tau concentration (or brain volumes) × time. The p-values are adjusted using false discovery rate (FDR). Beta (SE) refers to standardized regression coefficient with standard error. Cohen’s d refers to effect size of interaction effect of CSF t-tau concentration (or brain volumes) × time. In ADAS13, negative effect size d means higher volumes are correlated with slower ADAS13 score increase, whereas positive effect size d means higher baseline CSF t-tau concentration is correlated with faster ADAS 13 score increase. In MMSE, positive effect size d means higher volumes are correlated with slower MMSE score decrease.*

*Means the model did not fit well.

Then, we used structural MRI to evaluate specific brain regions atrophy. The LME model revealed that there was a significant interaction effect of adjusted hippocampal volume × time for ADAS13 in MCI A+ group (*p* = 0.04, β = –0.160) and MCI A+ combined AD A+ group (*p* = 0.04, β = –0.145; Table 6 and Figure 3C). However, this effect did not exist when we applied this model in the AD A+ group (*p* = 0.28, β = 0.148; Table 6). In CN groups (either Aβ negative or positive), adjusted hippocampal volume × time showed no significant interaction effect (*p* = 0.89, β = –0.012; *p* = 0.73, β = 0.083, respectively; Table 6). A similar LME model was performed using MMSE score to evaluate cognition and exhibited similar results (Table 6 and Supplementary Figure 3C).

Next, we evaluated the role of other brain regions, including the entorhinal and middle temporal lobe, in the longitudinal cognitive decline. Using the similar method as previously described, significant interaction effects of adjusted entorhinal volume × time, adjusted middle temporal lobe volume × time or adjusted whole brain volume × time were confirmed in MCI A+ combined AD A+ group rather than in CN A-, CN A+, or AD A+ groups (Table 6, Figures 3D–F, and Supplementary Figures 3D–F).

### The Comparison of Cerebrospinal Fluid and Plasma β Amyloid Deposition, Pathologic Tau, and Neurodegeneration [AT(N)] Biomarkers

We also compared the effect size of CSF sTREM2, p-tau181, and brain atrophy in predicting a cognitive decline, which was measured by Cohen’s d in the LME model. The effect size of CSF sTREM2 × time interaction was –0.60 in MCI A+ and AD A+ group and –0.58 in the MCI A+ group (where cognition was measured by ADAS13; Table 5), suggesting that it was a medium effect. The effect size of CSF p-tau181 × time interaction was –0.53 in MCI A+ and AD A+ group, and –0.56 in the MCI A+ group (Table 4), which is considered as a medium effect. While in cognitive impairment group, the effect size of the entorhinal, middle temporal lobe, and whole brain were 0.73, 0.72, and 0.72, respectively (cognition was measured by ADAS13), suggesting a larger effect size than CSF p-tau181 and CSF sTREM2. When cognition was measured by MMSE, we also discovered a larger effect size of brain volumes than CSF biomarkers (Table 6). Thus, CSF sTREM2 and p-tau181 might have a similar medium prediction effect for longitudinal cognition decline in cognitive impairment subjects with positive Aβ pathology, while brain volumes showed stronger effects in predicting cognition decline.

To assess whether plasma biomarkers could predict cognition change similar to CSF biomarkers, we applied the LME model of plasma t-tau, p-tau181, and Aβ42/Aβ40 ratio in a subgroup subject. These subjects were from previously studied 430 subjects and possessed baseline plasma biomarkers. A total of 233 subjects were enrolled from 430 subjects, including 53 CN subjects with negative amyloid, 26 CN subjects with positive amyloid, 64 EMCI subjects with positive amyloid, 52 LMCI subjects with positive amyloid, and 38 AD subjects with positive amyloid. No significant interaction effects of plasma t-tau and Aβ42/Aβ40 ratio in cognitive impairment groups were discovered (*p* = 0.59, β = –0.073; *p* = 0.79, β = 0.033; respectively). For plasma p-tau181, we confirmed higher plasma p-tau181 concentration could predict faster ADAS13 score change (*p* = 0.03, β = 0.123; Table 7). The effect size d of plasma p-tau181 concentration × time was 0.53 in cognitive impairment groups, suggesting a medium predicting effect. CSF p-tau181 had an effect size of 0.9, indicating a stronger effect than plasma p-tau181.

**TABLE 7:** Statistical analysis of linear mixed-effect regression for plasma biomarkers.

Plasma ratio	ADAS13	*P*	Adjusted *P*	Beta (SE)	*d*	MMSE	*P*	Adjusted *P*	Beta (SE)	*d*
CN A^–^		0.32	0.79	–0.147 (0.144)	NA		NA	NA	NA	NA
CN+MCI+AD A^+^		0.56	0.79	0.059 (0.099)	NA		0.42	0.46	–0.094 (0.117)	NA
MCI+AD A^+^		0.79	0.79	0.033 (0.125)	NA		0.46	0.46	–0.107 (0.143)	NA
**Plasma t-tau**	**ADAS13**	***P***	**Adjusted *P***	**Be ta (SE)**	***d***	**MMSE**	***P***	**Adjusted *P***	**Beta (SE)**	***d***
CN A^–^		0.59	0.59	0.065 (0.118)	NA		0.783	0.78	–0.036 (0.130)	NA
CN+MCI+AD A^+^		0.29	0.59	–0.083 (0.078)	NA		0.65352	0.78	–0.047 (0.105)	NA
MCI+AD A^+^		0.43	0.59	–0.073 (0.092)	NA		0.555	0.78	–0.071 (0.120)	NA
**Plasma p-tau181**	**ADAS13**	***P***	**Adjusted *P***	**Beta (SE)**	***d***	**MMSE**	***P***	**Adjusted *P***	**Beta (SE)**	***d***
CN A^–^		0.95	0.95	0.129 (0.106)	NA		0.23	0.23	–0.005 (0.085)	NA
CN+MCI+AD A^+^		0.01	0.03	0.120 (0.047)	0.51		0.01	0.04	–0.180 (0.070)	–0.53
MCI+AD A^+^		0.02	0.03	0.123 (0.052)	0.45		0.02	0.03	–0.174 (0.076)	–0.48

*p refers to the significance of interaction effect of plasma biomarkers concentration × time. The p-values are adjusted using false discovery rate (FDR). Beta (SE) refers to standardized regression coefficient with standard error. Cohen’s d refers to effect size of interaction effect of plasma biomarkers concentration × time. Positive effect size d means higher plasma p-tau are correlated with faster ADAS13 score increase. Negative effect size d means higher baseline plasma p-tau concentration is correlated with faster MMSE score decrease.*

## Discussion

AD is a devastating disease that brings a heavy burden to society and families. Reliable prognostic and outcome measures are of great importance in clinical practice and clinical trials. However, biomarkers used for predicting longitudinal cognitive change in the AD continuum are still unknown. Tau pathology, neuroinflammation, and neurodegeneration are the leading potential predictors for longitudinal cognitive change. However, the results of previous studies were controversial and still needed to be ascertained (Brier et al., 2016; Malpetti et al., 2020; Meyer et al., 2022; Morenas-Rodríguez et al., 2022; Pichet Binette et al., 2022). Moreover, rare studies have discussed their effect size of prediction role. In this study, we evaluated the ability of baseline CSF sTREM2, PGRN, p-tau181, Aβ42/40, t-tau, brain volumes, and plasma β amyloid deposition, pathologic tau, and neurodegeneration [AT(N)] biomarkers to predict the longitudinal cognitive deterioration in subjects with positive AD pathology. We also compared the effect size of these biomarkers in predicting longitudinal cognition decline for the first time.

Our study showed that baseline CSF sTREM2, CSF and plasma p-tau181, hippocampal volume, entorhinal volume, middle temporal lobe volume, and whole-brain volume could predict longitudinal cognitive decline. However, another key pathology Aβ-load measured by CSF and plasma Aβ42/Aβ40 ratio showed a negative correlation with longitudinal cognition change. Meanwhile, neurodegeneration (especially brain volumes) showed maximal prediction effect size while CSF p-tau181 and sTREM2 exhibited similar effect size. For plasma and CSF comparison, plasma p-tau181 showed a smaller effect size than CSF p-tau181 but still a medium effect. These findings extended the clinical use of CSF sTREM2 and plasma p-tau181.

Several studies have confirmed Aβ42/40 ratio was better in AD diagnosis and better concordance with Aβ PET than CSF Aβ42 (Janelidze et al., 2016, 2017), we used Aβ42/40 < 0.138 to define Aβ positive in this study. It has been reported that the aggregation of p-tau correlates with synaptic dysfunction and neuronal loss and is associated with the clinical severity of AD (Coomans et al., 2021). A research group demonstrated that subjects with higher postmortem autopsy confirmed neurofibrillary tangle (NFT) stage (Braak stage) exhibited a faster hippocampal volume change. In contrast, the Aβ stage (Thal stage) was not associated with longitudinal hippocampal atrophy (Josephs et al., 2017). One of the reasons is that p-tau burden increased in the cognitive impairment period while Aβ load reached a plateau in MCI and AD stage (Jack et al., 2010). These differences might explain how CSF p-tau could predict cognitive decline while CSF Aβ could not. Similarly, a recent study demonstrated that plasma p-tau181 was helpful in predicting AD-related neurodegeneration (Tissot et al., 2021).

Microglial activation is believed to be one of the core pathologies of AD. Some studies demonstrated that CSF sTREM2 exhibited a dynamic change during the AD progression (Suárez-Calvet et al., 2016; Ma et al., 2020). In this study, we did not find significant differences in baseline CSF sTREM2 in MCI or AD as compared with CN. However, higher baseline CSF sTREM2 predicted a slower longitudinal cognition decline. The baseline CSF concentration of another neuroinflammation biomarker, PGRN, showed no differences among CN, MCI, and AD group and did not have the ability to predict cognition decline. Although PGRN might act as a neuroinflammation and FTLD diagnostic biomarker, its role in diagnosing AD is controversial (Morenas-Rodríguez et al., 2016; Suárez-Calvet et al., 2018). Our results supported that CSF PGRN may not be a reliable diagnostic or prognostic biomarker for AD.

Next, we assessed the role of neurodegeneration biomarkers using CSF t-tau and brain volumes in predicting cognition decline. Our result showed that brain atrophy could predict cognitive change with a large effect size while CSF t-tau could not. These results coincided with several previous studies. A recent study showed that the cortical thickness and sulcus depth indices were significantly changed during AD progression, suggesting that gray matter characteristics can track AD progression (Wu et al., 2021). Similarly, another study demonstrated distinct patterns of gray matter atrophy associated with mild behavioral impairment in CN subjects (Shu et al., 2021). In addition, hippocampal, entorhinal, middle temporal lobe, and whole-brain volume showed a significant decrease during the disease progression, indicating brain atrophy occurred in the whole disease period. However, statistical results did not exhibit a smaller hippocampal volume in AD compared to CN and MCI groups, which might result from insufficient subjects when we used 1.5 T or 3.0 T MRI data alone. These results identified neurodegeneration as a major factor in the disease progression and could be used as prognostic measurements for AD.

Finally, we evaluated the differences between CSF and plasma biomarkers in predicting cognition decline. We discovered plasma Aβ42/Aβ40 ratio and t-tau were not correlated with longitudinal cognitive decline while plasma p-tau181 predicted longitudinal cognitive decline. Recent studies confirmed that plasma Aβ42/Aβ40 ratio was correlated with PET measured brain amyloid deposition. However, due to different environment, plasma Aβ42/Aβ40 ratio was less prominent in predicting amyloidosis compared to CSF Aβ42/Aβ40 ratio and shows a larger overlap between subjects with PET measured amyloid-positive and amyloid-negative (Ovod et al., 2017; Nakamura et al., 2018). Similar to the plasma Aβ42/Aβ40 ratio, a study demonstrated that plasma t-tau was less specific to discriminate several neurodegenerative diseases and shows fewer correlations with clinical severity than plasma neurofilament light chain (NFL; Illán-Gala et al., 2021). In contrast, plasma p-tau181 was more specific in diagnosing AD, predicting brain tau and Aβ pathology and disease severity (Moscoso et al., 2021). A previous study discovered that CSF and plasma p-tau181 are correlated in Aβ-positive subjects rather than in Aβ-negative subjects. Plasma p-tau181 shows discrimination accuracy similar to CSF p-tau181 (Janelidze et al., 2020). As a result, plasma-tau181 tightly correlates with disease severity rather than Aβ42/Aβ40 ratio and plasma t-tau. These might explain the diverse role of plasma p-tau181, Aβ42/Aβ40 ratio, and t-tau in predicting longitudinal cognition decline. However, a recent study demonstrates that individuals with low plasma Aβ42/40 experience faster cognitive decline.

Rare studies have compared the role of CSF and plasma p-tau181 in predicting longitudinal cognitive decline. In this study, we found that plasma p-tau181 had a smaller effect than CSF p-tau181. As plasma p-tau181 was derived from CSF p-tau181, and there is a different environment between peripheral circulation and central nervous system, it is not surprising that plasma and CSF p-tau showed a bit different effect size. Nevertheless, our study supported that plasma p-tau181 can be used as a reliable relatively noninvasive biomarker for longitudinal cognition decline prediction as higher baseline plasma p-tau181 predicted faster longitudinal cognitive decline.

There were several limitations in this study. First, most of the AD subjects only had a follow-up visit for 2 years and did not have enough longitudinal ADAS13 and MMSE scores, which might affect the results of the LME model computed in the AD group (Supplementary Table 1). Hence, studies with more longitudinal cognitive change data were needed in the future. Second, a number of biomarkers can reflect brain neuroinflammation. This study only selected CSF sTREM2 and PGRN. More other measurements are needed to confirm our results.

Collectively, our study reported that baseline CSF sTREM2, CSF, and plasma p-tau181, as well as structural MRI, could predict longitudinal cognitive decline in subjects with positive AD pathology. CSF sTREM2, CSF, and plasma p-tau181 had similar medium prediction effect, while brain volumes showed stronger effects in predicting cognition decline. Plasma p-tau181 can be used as a reliable relatively noninvasive biomarker for longitudinal cognition decline prediction.

## Data Availability Statement

The original contributions presented in this study are included in the article/Supplementary Material, further inquiries can be directed to the corresponding author.

## Ethics Statement

The studies involving human participants were reviewed and approved by regional ethics committees of all institutions joined in ADNI. The patients/participants provided their written informed consent to participate in this study.

## Author Contributions

Y-HC: analysis of the data and drafting the original manuscript. R-RL, H-FH, and Y-YX: data acquisition and analysis. Q-QT: funding, study design, and critical revision of the manuscript. All authors have contributed to the article, revision of the manuscript, and approved the final manuscript.

## Conflict of Interest

The authors declare that the research was conducted in the absence of any commercial or financial relationships that could be construed as a potential conflict of interest.

## Publisher’s Note

All claims expressed in this article are solely those of the authors and do not necessarily represent those of their affiliated organizations, or those of the publisher, the editors and the reviewers. Any product that may be evaluated in this article, or claim that may be made by its manufacturer, is not guaranteed or endorsed by the publisher.
