# Supplementary material for: Microglial Activation, Tau Pathology, and Neurodegeneration Biomarkers Predict Longitudinal Cognitive Decline in Alzheimer’s Disease Continuum
Source: Front Aging Neurosci. 2022 Jun 30;14:848180. doi: 10.3389/fnagi.2022.848180 (PMC9280990; doi:10.3389/fnagi.2022.848180)
Supplement: Supplementary file 1 [file Data_Sheet_1.docx]

**Supplementary Materials**

**Supplementary Figure 1** Baseline brain volumes (segmented in 1.5 T structural MRI) comparison between CN A-, CN A+, MCI A+ and AD A+ groups. ***<0.001,**<0.01,*<0.05,ns: not significant.

**
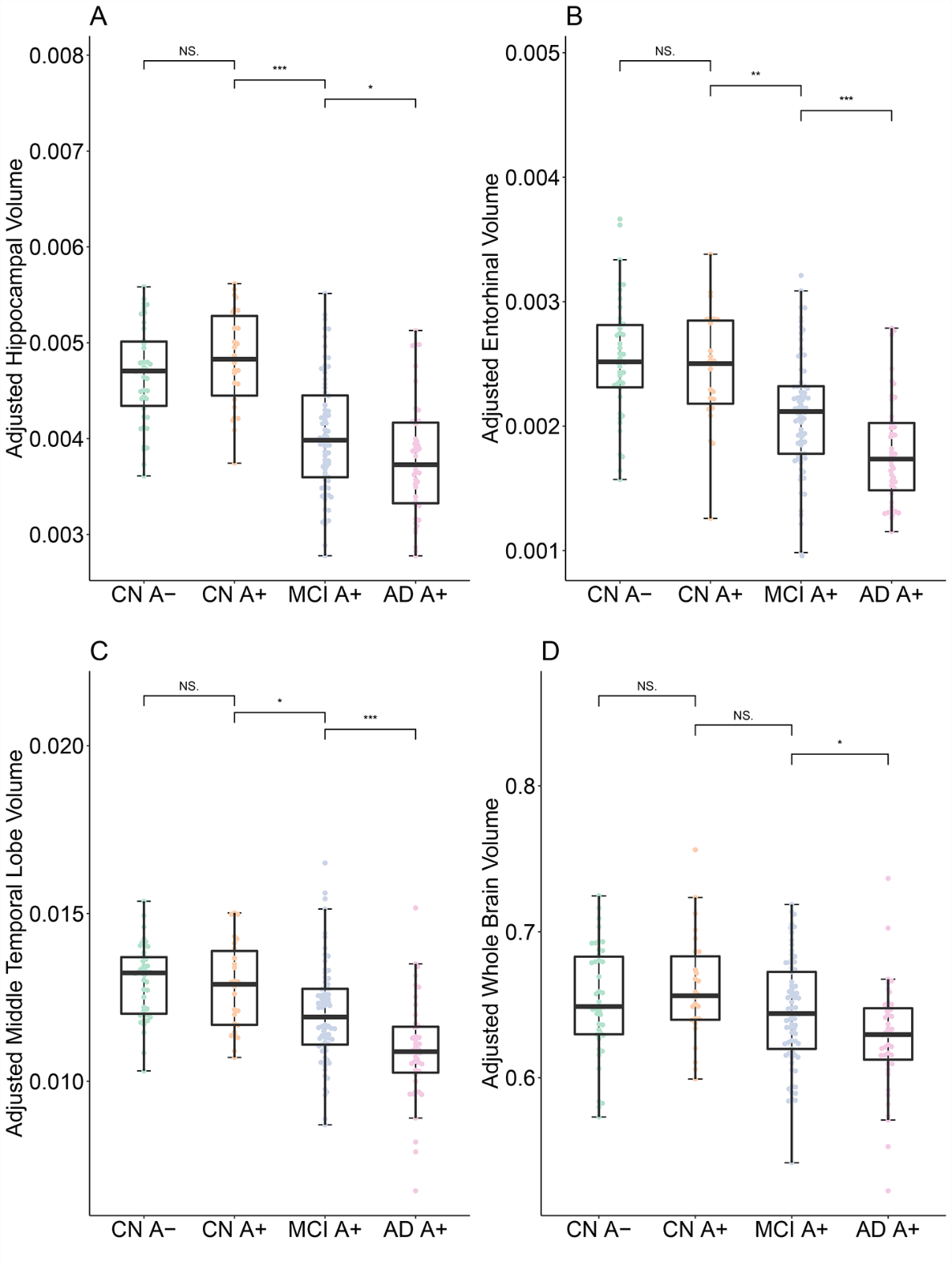
**

**Supplementary Figure 2** Correlation of baseline brain volumes with cognition status. A-D is the regression plot of brain volumes. Note that the brain volumes were segmented from 1.5T structural MRI. **
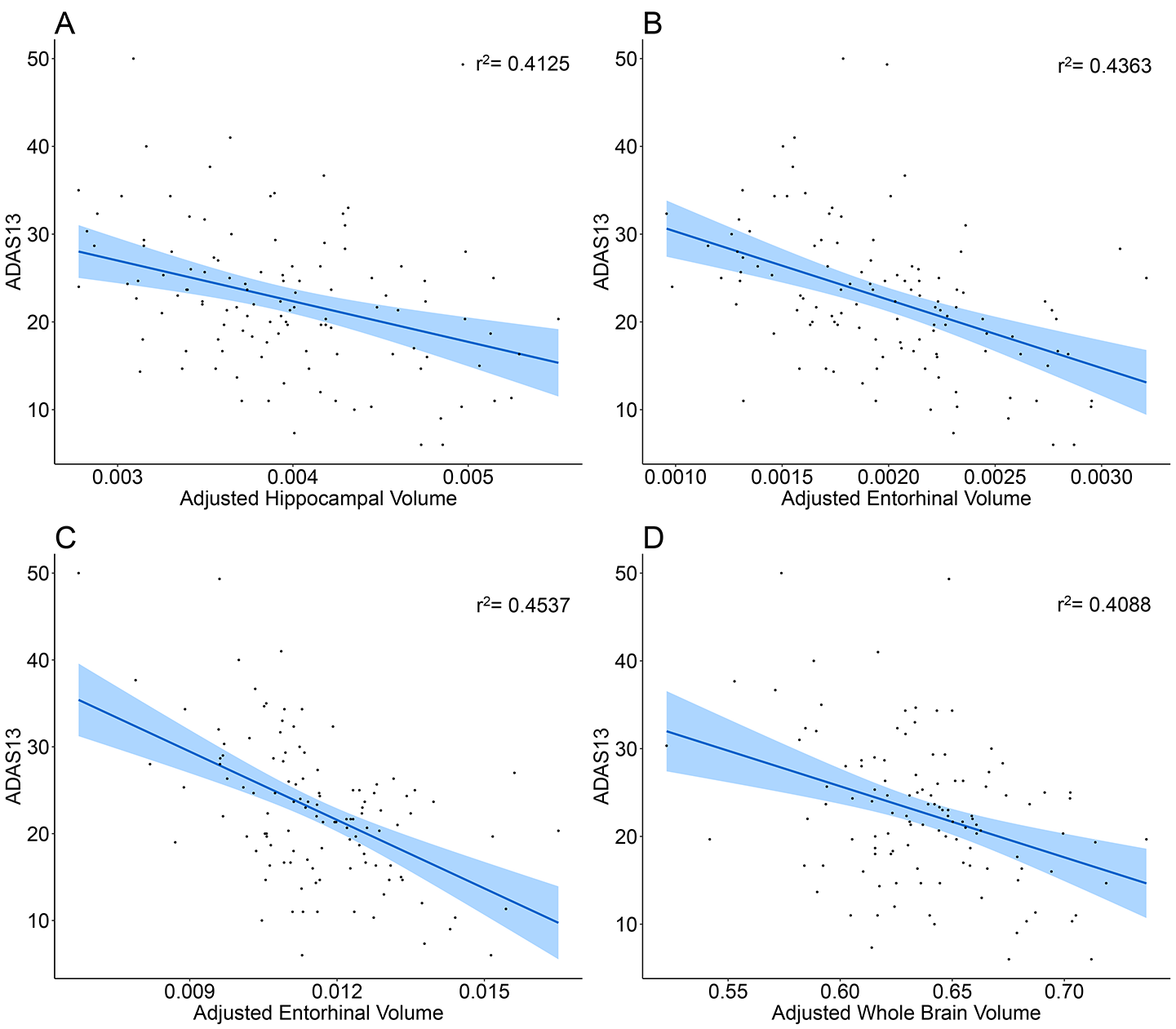
**

**Supplementary Figure 3** Role of each biomarker in cognitive change measured by MMSE. A-F show the annual MMSE change in cognitive impairment population with positive Aβ pathology. For directly illustration, each plot compares ADAS scores between the sub-group who scored below the median (in red) and the sub-group who scored above the median (in green) on the variable in the plot title. Brain volumes are segmented from 3.0T structural MRI.


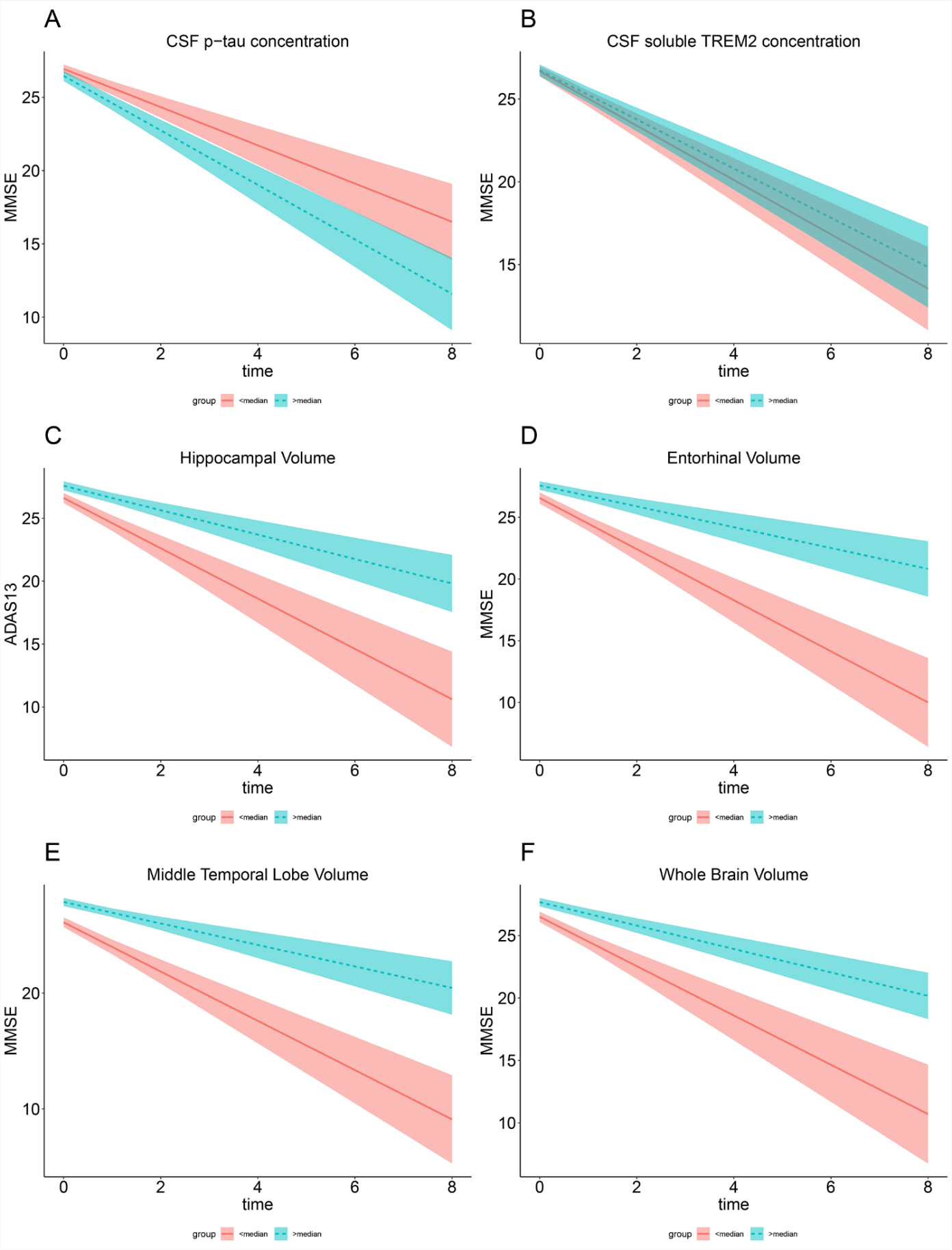


**Supplementary Table 1** The number of cognitive measurements in each visit time point (M12=month 12).

| Visit time points | Baseline  (ADAS13/MMSE) | M12 | M24 | M36 | M48 | M60 | M72 | M84 | M96 |
| --- | --- | --- | --- | --- | --- | --- | --- | --- | --- |
| CN A- | 96/96 | 94/95 | 91/91 | 43/43 | 61/62 | 31/31 | 51/51 | 29/28 | 33/33 |
| CN A+ | 54/54 | 52/52 | 53/53 | 26/26 | 40/40 | 17/18 | 27/27 | 17/17 | 15/16 |
| MCI A+ | 195/195 | 194/167 | 166/167 | 135/136 | 84/85 | 55/58 | 41/43 | 33/35 | 22/22 |
| AD A+ | 85/85 | 83/84 | 49/53 | 5/7 | 2/2 |  |  |  |  |

**Supplementary Table 2** Follow up data of cognitive assessments measured by ADAS13 and MMSE (data were shown in mean±sd).

| Visit time points | M12 | M24 | M36 | M48 | M60 | M720 | M84 | M96 |
| --- | --- | --- | --- | --- | --- | --- | --- | --- |
| ADAS13 | | | | | | | | |
| CN A- | 8.43±4.27 | 8.54±4.10 | 8.81±4.50 | 8.62±4.18 | 9.61±3.41 | 12.07±5.38 | 11.58±6.48 | 11.91±4.42 |
| CN A+ | 8.52±4.85 | 9.10±5.50 | 10.22±6.67 | 10.27±5.45 | 11.94±6.55 | 13.77±4.76 | 16.33±7.66 | 18.02±7.29 |
| MCI A+ | 19.48±8.33 | 21.47±10.41 | 24.06±12.71 | 26.07±15.94 | 26.68±13.08 | 28.84±15.10 | 30.19±12.57 | 35.44±15.74 |
| AD A+ | 35.92±10.55 | 38.50±4.21 | 34.73±6.74 | 31±6.67 |  |  |  |  |
| MMSE | | | | | | | | |
| CN A- | 28.94±1.22 | 29.24±1.20 | 28.63±1.49 | 29.29±1.05 | 29.39±1.01 | 28.88±1.69 | 28.82±1.56 | 29.21±1.09 |
| CN A+ | 28.71±1.61 | 28.66±1.21 | 28.73±1.16 | 28.38±1.87 | 27.61±2.56 | 28.04±2.03 | 27.24±2.16 | 27±2.35 |
| MCI A+ | 26.36±2.93 | 25.80±3.36 | 24.40±4.90 | 24.46±5.76 | 23.60±5.35 | 22.65±7.01 | 23.2±6.39 | 20.5±6.60 |
| AD A+ | 20.21±4.50 | 18.17±5.20 | 15.71±9.04 | 21±4 |  |  |  |  |

**Supplementary Table 3** Correlations of baseline brain volumes (segmented in 1.5T structural MRI) with cognitive status (measured by ADAS13) in subjects with cognition impairment.

|  | *P* | Beta(SE) | R^2^ |
| --- | --- | --- | --- |
| Adjusted HV | 0.000661 | -4.010e+03 (1.143e+03) | 0.4125 |
| Adjusted EV | 0.0000633 | -5.762e+03 (1.383e+03) | 0.4363 |
| Adjusted MTLV | 0.0000109 | -1.827e+03 (3.957e+02) | 0.4537 |
| Adjusted WBV | 0.000945 | -63.70540 (18.73114) | 0.4088 |

The correlations of brain volumes with cognitive status (measured by ADAS13) were analyzed using multivariate linear regression. Beta (SE) refers to standardized regression coefficient with standard error. R^2^ refers to coefficient of determination. HV=Hippocampal volume; EV=Entorhinal volume; WBV=Whole brain volume MTL=Middle temporal lobe volume.

**Supplementary Table 4** Statistical analysis of linear mixed effect regression for specific brain region volumes (segmented in 1.5T structural MRI) in amyloid positive (A^+^) groups alone or in combination.

| Adjusted HV | ADAS13 | *P* | Adjusted *P* | Beta (SE) | d | MMSE | *P* | Adjusted *P* | Beta (SE) | d |
| --- | --- | --- | --- | --- | --- | --- | --- | --- | --- | --- |
| CN A^-^ |  | 0.03 | 0.08 | -0.241 (0.107) | -0.86 |  | 0.47 | 0.47 | 0.089 (0.123) | NA |
| CN A^+^ |  | 0.03 | 0.08 | -0.350 (0.146) | NA |  | 0.16 | 0.32 | 0.223 (0.153) | NA |
| MCI A^+^ |  | 0.41 | 0.51 | -0.093 (0.112) | NA |  | 0.049 | 0.20 | 0.255 (0.127) | 0.55 |
| AD A^+^ |  | 0.24 | 0.40 | 0.116 (0.095) | NA |  | NA^*^ | NA | NA | NA |
| MCI+AD A^+^ |  | 0.87 | 0.87 | -0.015 (0.090) | NA |  | 0.28 | 0.37 | 0.118 (0.110) | NA |
| Adjusted EV | **ADAS13** | ***P*** | **Adjusted *P*** | **Beta (SE)** | **d** | **MMSE** | ***P*** | **Adjusted *P*** | **Beta (SE)** | **d** |
| CN A^-^ |  | 0.21 | 0.26 | -0.143 (0.110) | NA |  | 0.47 | 0.63 | 0.090 (0.122) | NA |
| CN A^+^ |  | 0.004 | 0.02 | -0.436 (0.137) | NA |  | 0.95 | 0.95 | 0.011 (0.171) | NA |
| MCI A^+^ |  | 0.06 | 0.15 | -0.176 (0.091) | NA |  | 0.02 | 0.08 | 0.249 (0.105) | 0.61 |
| AD A^+^ |  | 0.26 | 0.26 | 0.150 (0.129) | NA |  | NA^*^ | NA | NA | NA |
| MCI+AD A^+^ |  | 0.09 | 0.15 | -0.133 (0.077) | NA |  | 0.046 | 0.092 | 0.190 (0.094) | 0.43 |
| Adjusted MTLV | **ADAS13** | ***P*** | **Adjusted *P*** | **Beta (SE)** | **d** | **MMSE** | ***P*** | **Adjusted *P*** | **Beta (SE)** | **d** |
| CN A^-^ |  | 1.0 | 1.0 | 0.000 (0.116) | NA |  | 0.08 | 0.11 | 0.216 (0.119) | NA |
| CN A^+^ |  | 0.31 | 0.39 | -0.164 (0.155) | NA |  | 0.19 | 0.19 | 0.211 (0.153) | NA |
| MCI A^+^ |  | <0.001 | <0.001 | -0.364 (0.099) | -0.91 |  | 0.003 | 0.006 | 0.371 (0.118) | 0.82 |
| AD A^+^ |  | 0.014 | 0.02 | -0.242 (0.093) | -1.0 |  | NA^*^ | NA | NA | NA |
| MCI+AD A^+^ |  | <0.001 | 0.001 | -0.397 (0.077) | -1.1 |  | <0.001 | <0.001 | 0.501 (0.095) | 1.1 |
| Adjusted WBV | **ADAS13** | ***P*** | **Adjusted *P*** | **Beta (SE)** | **d** | **MMSE** | ***P*** | **Adjusted *P*** | **Beta (SE)** | **d** |
| CN A^-^ |  | 0.58 | 0.58 | -0.067 (0.120) | NA |  | 0.24 | 0.32 | 0.155 (0.128) | NA |
| CN A^+^ |  | 0.30 | 0.38 | -0.186 (0.174) | NA |  | 0.79 | 0.79 | 0.047 (0.173) | NA |
| MCI A^+^ |  | 0.03 | 0.05 | -0.222 (0.102) | -0.55 |  | 0.008 | 0.02 | 0.317 (0.116) | 0.74 |
| AD A^+^ |  | 0.03 | 0.05 | -0.218 (0.096) | NA |  | NA^*^ | NA | NA | NA |
| MCI+AD A^+^ |  | 0.002 | 0.01 | -0.255 (0.081) | -0.67 |  | <0.001 | <0.001 | 0.395 (0.096) | 0.89 |

## *P* refers to the significance of interaction effect of regional brain volumes × time. P values are adjusted using FDR (false discovery rate). Beta (SE) refers to standardized regression coefficient with standard error. Cohen’s d refers to effect size of interaction effect of regional brain volume × time. In ADAS13, negative effect size *d* means higher volumes are correlated with slower ADAS13 score increase, whereas positive effect size *d* means higher baseline CSF t-tau concentration is correlated with faster ADAS 13 score increase. In MMSE, positive effect size d means higher volumes are correlated with slower MMSE score decrease. *Means the model did not fit well.
